# Supplementary figures and images for: Those who tan and those who don’t: A natural experiment on colorism
Source: PLoS One. 2020 Jul 24;15(7):e0235438. doi: 10.1371/journal.pone.0235438 (PMC7380621; doi:10.1371/journal.pone.0235438)

**S1 Appendix**

Figure 1A: The Fitzpatrick Scale


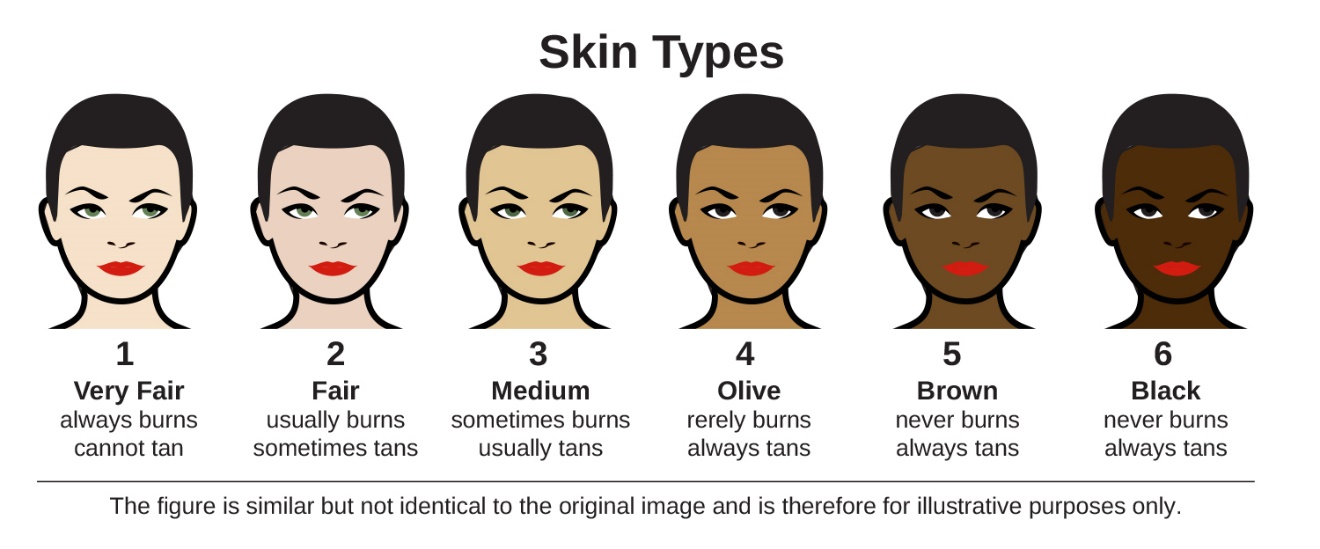

Supplement: S1 Appendix — (DOCX) [file pone.0235438.s001.docx]
